# Stress Due to Inflation: Changes over Time, Correlates, and Coping Strategies among Working-Age Adults in the United States

**DOI:** 10.3390/ijerph21020157

**Published:** 2024-01-30

**Authors:** Sophie Mitra, Chan Shen, Jahnavi Pinnamraju, R. Constance Wiener, Hao Wang, Mona Pathak, Patricia A. Findley, Usha Sambamoorthi

**Affiliations:** 1 Department of Economics, Fordham University, 441 East Fordham Road, Bronx, NY 10458, USA; 2 Department of Surgery, Penn State Cancer Institute, Hershey, PA 17033, USA; 3 Department of Pharmacotherapy, College of Pharmacy, University of North Texas Health Science Center, Fort Worth, TX 76107, USA; 4 Department of Dental Public Health and Professional Practice, West Virginia University, Morgantown, WV 26506, USA; 5 Department of Emergency Medicine, JPS Health Network, Integrative Emergency Services, Fort Worth, TX 76104, USA; 6 School of Social Work, Rutgers University, New Brunswick, NJ 08901, USA

**Keywords:** stress due to inflation, social determinants of health

## Abstract

Background: During the COVID-19 pandemic, the annual US inflation rate increased from 1.2% in July 2020 to 8% in July 2022. It has since declined to 3.4% (December 2023). This study examined the prevalence of stress due to inflation during a period when it declined from 8.2% in September 2022 to 3% in June 2023 and its association with demographic and social determinants of health (SDOH). Methods: We conducted a cross-sectional analysis of the online Household Pulse Survey (HPS), which surveils the experiences of US households. Beginning September 2022, HPS initiated data collection on “stress due to inflation” through a question on how stressful the increase in prices in the last 2 months has been. Participants could respond: very, moderately, a little, or not stressful. We analyzed data on working-age adults (18–64 years) who responded to the above question of stress due to inflation during 14–26 September 2022 (N = 32,579) and 7–19 June 2023 (N = 36,229). We used replicate weights in chi-squared tests and ordinal logistic regression analyses controlling for gender, age, race and ethnicity, COVID-19, COVID-19 vaccination, health insurance, and SDOH, including education, lost employment income, poverty status, marital status, food affordability, and region. Results: The prevalence of stress due to inflation (price increases being very or moderately stressful) significantly increased from 76.9% in September 2022 to 78.9% in June 2023. The odds of stress due to inflation were higher for individuals with the following characteristics: female, transgender, having income below 400% of the federal poverty line, having lost employment income, not being able to afford food, had long or acute COVID-19, and did not have a COVID-19 vaccine. Conclusions: More than three quarters of working-age adults in the US experienced stress due to inflation. Despite a declining national inflation rate in recent months, stress due to inflation has significantly increased among working-age adults. Inflation-related stress warrants further research and policy attention.

## 1. Introduction

During the COVID-19 pandemic, the annual inflation rate in US increased from 1.2% in 2020 to 8% in 2022 [1,2]. Historically, since 1960, the inflation rate in the US has been low, although there was a major rise in the 1970s that led to a high of 13.6% in 1980 [2]. From early 2021, the inflation rate in the US gradually increased, peaked at 9.1% in July 2022, and declined to 3.4% as of December 2023 [3].

Inflation reduces the purchasing power of money and increases everyone’s cost of living. It can constrain the spending patterns of some individuals and households, and may make meeting basic needs and well-being difficult [4]. Inflation may be viewed as a threat [5,6] to maintaining a standard of living or to meeting basic needs (e.g., food, housing). Research has supported the existence of a response of “psychological stress” to an external threat [7]. When faced with an external threat, the survival stress response (fight, flight, or freeze) is initiated, which may partly explain the relationship between inflation and stress due to inflation [8]. Inflation may be thought of as a macrolevel economic stressor [9] that many US residents have had little exposure to, and thus has rarely been measured and researched.

A few researchers from the 1980s [10] and in the past year [4,5] have measured stress due to inflation. They found that for some individuals and families, inflation may make meeting basic needs difficult, as inflation interrelates with other stressors such as poverty, job loss, and/or discrimination. These factors often lead to high levels of stress and reductions in well-being [4]. In addition, despite declining inflation in recent months, stress due to inflation might persist and may become chronic (lasting six months or more) [11]. While inflation rates have recently decreased, they are not negative, so price levels continue to be historically high and to increase [12]. As a stressor, inflation has the potential to impact both physical and mental health. For instance, stress is associated with greater risk of cardiovascular disease, hypertension, and infectious disease [11].

It is possible that there are differences between objective measures of inflation and people’s perceptions of inflation. Individuals may not revise their perceptions in real time in line with the inflation measure updates provided by reporting agencies or news media. However, consumers may revise their perception of inflation based on current prices, although these adjustments may take longer periods of time [13,14,15]. Furthermore, there may be subgroup variations in the prevalence of stress due to inflation. Individuals with current or expected financial difficulties often have a larger upward bias in their expectations of inflation than others [16], which may lead to subgroup differences in perceptions of stress due to inflation and its persistence.

This study fills a knowledge gap by taking advantage of new and current data on perceptions of stress due to inflation from the Household Pulse Survey (HPS), which the US Census Bureau began collecting in 2022 [17]. Importantly, taking advantage of data from both September 2022 and June 2023, during which the inflation rate decreased from 8.2% to 3%, this study considers the dynamics between a declining inflation rate and stress due to inflation. Further, the study of coping mechanisms adds to the emerging literature on the prevalence of stress due to inflation since the COVID-19 pandemic. A recent letter, also using HPS data, identified inflation as a significant source of stress among all adults aged 18 years and above in the US, especially among women and socioeconomically vulnerable groups [4].

In this study, we focus on working-age adults (age ≥ 18 to 64 years) because of major differences in the economic situation of working-age adults and older adults. Older adults in the US often have access to Social Security benefits with cost-of-living adjustment, which ensures that the purchasing power of the benefit amount may not be affected inordinately by inflation or price increases. In contrast, many working-age adults may have been unemployed or underemployed during the pandemic or have earnings that match or exceed the inflation rate. There was a loss of 21 million jobs from the fourth quarter of 2019 to the second quarter of 2020, and during that time, the unemployment rate rose from 3.6% to 13.0% [18]. Although resilient working-age adults may have many opportunities, the loss of wages and economic insecurity may lead to feelings of vulnerability associated with price increases. Furthermore, a majority (64%) may be living paycheck to paycheck [19] and average older adults are wealthier than the working-age adults aged 35 years [20]. Therefore, many working-age adults may not have savings as a financial cushion against inflation.

It is also unclear whether stress due to inflation goes down when the national inflation rate declines and if it persists or even increases due to price levels remaining high over time. Although inflation rates above 5% were short-lived in the past in the US, we hypothesize that stress due to inflation after COVID is persistent, potentially increasing, and not sensitive to recent declines in the inflation rates, given continually high price levels that many individuals and families have had little previous experience with and find difficult to manage. The previous low annual inflation rates for a very long time (about 40 years) might have left many unprepared for this economic challenge. As strategies to cope with inflation may involve behavior changes that may be negative or positive, we also describe broad categories of coping behaviors.

Therefore, the primary objective of this study is to analyze stress due to inflation over time and factors associated with stress due to inflation among working-age adults. To better understand the stress due to inflation, including its persistence, correlates, and coping strategies, we analyzed and compared cross-sectional data from two time periods collected by the Census Bureau, 14–26 September 2022 and 7–19 June 2023, when the inflation rate declined from 8.2% to 3% respectively.

## 2. Methods

Study design: We conducted a pooled cross-sectional analysis of data from the online Household Pulse Survey (HPS) collected between 14–26 September 2022 and 7–19 June 2023.

Data Source: The HPS is conducted by the Census Bureau in coordination with 16 other federal agencies to examine the impact of coronavirus on the social and economic outcomes as well as other emergent issues in American households. The HPS is designed to be deployed quickly and efficiently, and therefore the survey is administered frequently, once every week until September 2021 and once every two weeks since October 2021 [21]. In September 2022 (the bi-week identified as week 49 by HPS), the HPS began collecting information on individuals’ perceptions of price changes in the areas they live in the last two months, as well as how the price increases have affected the US households, which we refer to as stress due to inflation throughout this manuscript [17].

### 2.1. Analytical Sample

The study was restricted to working-age adults who had reported that prices have increased in the past two months in the areas in which they live (N = 32,579 in September 2022 and N = 38,229 in June 2023). Participants were only interviewed once. Participants with missing responses to the question “How stressful, if at all, has the increase in prices in the last two months been for you?” were excluded (N = 250 in September 2022, and N = 380 in June 2023). The study sample included 70,808 working-age adults aged 18 to 64 years in the US in total, with 32,579 surveyed during 14–26 September 2022 and 38,229 surveyed during 7–19 June 2023.

### 2.2. Measures

Dependent Variable: The dependent variable, stress due to inflation, was measured using the following question: How stressful, if at all, has the increase in prices in the last 2 months been for you? Participants could respond: very, moderately, a little or not stressful. The responses in the original four categories were used as a dependent variable in the ordinal logistic regression.

Key Explanatory Variable: The key explanatory variable was the time period (14–26 September 2022 and 7–19 June 2023) surveyed. This study examined changes in stress due to inflation by taking advantage of the repeated administration of the survey with variables related to stress and coping strategies.

### 2.3. Other Explanatory Variables

Other explanatory variables included demographics (age groups in years (18–34, 35–44, 45–54, 55–64)), sex (female, male, transgender), race and ethnicity (Hispanic/Latino, non-Hispanic Asian, Non-Hispanic black, non-Hispanic white, and other). The SDOH considered were education (less than high school, high school, some college, and college), lost employment income by the respondent or any household member (yes or no), poverty status (with four categories ranging from <100%, 100–<200%, 200–<400%, >=400%), food affordability (yes or no), health insurance coverage (yes or no), marital status (married, widowed, separated, divorced, and never married), and region (Northeast, South, Midwest, West). We also included whether individuals had experienced COVID (long COVID, acute COVID, no COVID) [22,23] and receipt of COVID vaccination (yes or no) [24], as explanatory variables because these may exacerbate stress due to inflation.

### 2.4. Statistical Analysis

The statistically significant associations of categorical variables with stress due to inflation were examined with Rao–Scott chi-squared tests. We employed ordinal logistic regression for the stress level variable with four categories ranging from “very stressful” to “not at all stressful” to find the magnitude of the association of the time period with stress due to inflation while controlling for other explanatory variables. The relationship of time with stress due to inflation was analyzed for each subgroup separately (e.g., men, women and transgender separately). The odds ratios are represented as cumulative odds ratios. SAS survey procedures incorporating the jackknife method with replicate survey weights were used in all our analyses [25].

## 3. Results

The characteristics of working-age adults in the US in each time period are provided in Table 1. In both time periods, more than three quarters of the individuals surveyed reported being very/moderately stressed, and less than a quarter reported having little or no stress. When we compare September 2022 with June 2023, the chi-squared test suggests that there was a significant increase in the percentage who was very/moderately stressed due to inflation from 76.9% in September 2022 to 78.9% (*p* < 0.0001). We also observed statistically significant differences in gender, age, education, region and COVID status between the two time periods (Table 1).

Table 2 summarizes the characteristics of individuals by level of stress due to inflation during both time periods. We did observe subgroup differences in levels of stress due to inflation. The transgender group was more often very/moderately stressed due to inflation (85.8%) than females (80.9%) and males (74.3%). A higher percentage of Hispanic individuals were more very/moderately stressed due to inflation (82.6%) than non-Hispanic Blacks (81.3%), non-Hispanic Whites (76%) and non-Hispanic Asians (69.9%).

All SDOH were associated with stress due to inflation. In terms of marital status, the divorced, widowed, and separated groups had the higher prevalence of stress (all above 80%) compared to never married and married (both below 80%). College-educated individuals had the lowest prevalence of stress due to inflation at 66.5%, and all other groups had much higher stress prevalence at above 80%. Food affordability was highly associated with stress due to inflation, with 96.4% of individuals not being able to afford food reporting stress. Similarly, having health insurance was associated with lower stress (76.9% among insured compared to 83.2% among uninsured). Working-age adults with long COVID had a significantly higher rate of stress at 86.3% compared with those with no long COVID (73.6%) and no COVID (78.2%).

The multivariable ordinal logistic regression results are presented in Table 3. The cumulative odds ratio (COR) for experiencing stress due to inflation in June 2023 compared to September 2022 showed a statistically significant difference (COR = 1.08, 95% CI = 1.02, 1.14,). Female and transgender groups had higher odds of stress due to inflation than males (COR = 1.34, 95% CI = 1.27, 1.42, COR = 1.46, 95% CI = 1.22, 1.75). Race/ethnicity was not significantly associated with higher odds of stress.

Regarding SDOH, lost employment income was associated with higher odds of stress due to inflation (COR = 1.72, 95% CI = 1.53, 1.94). Poverty or having an income below 400% of the federal poverty line was associated with reporting more stress due to inflation. The inability to afford food had the highest COR in the model at 7.14 with a 95% CI of 6.58–7.74. Receiving the COVID-19 vaccine was associated with a lower likelihood of stress due to inflation (COR = 0.71, 95% CI = 0.65, 0.77), while long and acute COVID heightened the likelihood of stress due to inflation (COR = 1.46, 95% CI = 1.32, 1.62 and COR = 1.10, 95% CI = 1.03, 1.17).

As a sensitivity analysis, we ran the ordinal logistic model within each of the sub-groups of Table 2 (e.g., for men, transgender and women separately) (results are available from authors upon request). For several subgroups, stress significantly increased between September 2022 and June 2023: females, non-Hispanic Asians, adults aged 35–44, college-educated adults, adults who lost employment income, adults who cannot afford food, adults who were never married and those living in the South, while for one subgroup, adults who received COVID 19 vaccination, stress due to inflation significantly declined. For all other subgroups, there was no significant change over time in stress due to inflation.

The HPS also asked the respondents “What changes, if any, have you made or do you plan to make to cope with the increase in prices?” We analyzed these coping strategies, including by comparing them among working adults in September 2022 and those in June 2023 (Table 4). The respondents were given 19 options [23]. More than 90% of working-age adults make changes to cope with price increases. The most common coping behaviors adopted by more than half of working-age adults include shopping with low-price coupons, eating out less and delaying major purchases. Ten other coping behaviors were adopted by between a fifth and a half of the sample: switching from brand to generics, canceling/reducing subscriptions, canceling/reducing events, saving less, eating less meat and fresh produce, driving less/changing mode of transportation, decreasing the use of utilities, increasing the use of credit cards, delaying medical treatment, and working additional jobs.

Table 4 also shows how these changes in behaviors changed from September 2022 to June 2023. There was a significant decrease in the percentage of adults who did not make any changes to cope with the price increases (6.3% in June 2023 7.3% in September 2022 (*p* = 0.005)). Among the other 18 options, only four (i.e., saving less, canceling, or reducing events, decreasing the use of utilities, and seeking help from friends or family) showed no statistically significant difference over time (September 2022 versus June 2023). The use of the other three coping strategies (eating less meat, driving less or changing transportation modes, and increasing credit card usage) decreased from September 2022 to June 2023. Compared to September 2022, a higher percentage of working-age adults in June 2023 shopped for lower prices or using coupons, switched from brand names to generic products, ate out less, canceled/reduced subscriptions, delayed major purchases, delayed medical treatment, took on additional jobs, moved to a less expensive house, reduced childcare costs, utilized benefits for charities, and others.

From September 2022 to June 2023, individuals continued to make efforts to increase their incomes (e.g., seeking additional jobs, which may result in increased transportation costs) and reduce their expenses (e.g., eating out less, shopping for lower prices or using coupons, delaying medical treatment, or canceling or reducing subscriptions, to name a few). However, even with these various coping strategies, savings did not appear to change over time (36.9% reported less savings in September 2022 compared to 36.1% in June 2023, *p* = 0.418, Table 4).

## 4. Discussion

In this study, we found that stress due to inflation was very common among working-age adults, with more than three quarters reporting being very stressed or moderately stressed regarding price increases in the past two months. We also observed that despite declining inflation during 14–26 September 2022 and 7–19 June 2023, the prevalence of stress due to inflation increased significantly.

Although overall inflation is falling, the cost of food and groceries have remained high [26]. A majority of Americans (64%) live paycheck to paycheck [27]; the reduction in purchasing power to buy food and groceries, and historically high prices may explain increasing stress due to inflation.

One important characteristic of individuals who reported stress due to inflation was the belief that prices will continue to increase in the next six months (results not reported in tabular form). An overwhelming majority (78.4%) of working-age adults who were very stressed about price increases expected the prices to further increase in the next six months. Taken together, these findings suggest that perception indicators on stress due to inflation may not move together with the national inflation rate. In the literature on consumer inflation expectations, many have found that there are large variations in the perception and expectation of inflation [28,29], which may lead to subgroup differences in stress due to inflation. Researchers have also explained this as part of “issue-attention cycle” and “rational inattention”, in which it may take longer for perceptions of inflation to match the objective measures [30].

We also observed that working-age adults with unfavorable SDOH were more likely to experience stress due to inflation than others. For example, the odds of reporting stress due to inflation were higher among individuals with the inability to afford food. Our study findings suggest that disparities exist between individuals with and without stress due to inflation. Those who experience financial hardships such as the loss of employment income are more likely to be stressed due to inflation. These findings overall are consistent with a recent published letter, which used the HPS data in September 2022 to February 2023 [4] and found that stress due to inflation was higher among low-income households.

In addition, we found that working-age adults with COVID had higher odds of stress due to inflation compared to those without COVID. Such findings could probably, in part, be explained as COVID may have affected financial vulnerability, changing some individuals’ economic activities and their financial behaviors [31,32]. COVID affected more females and poor families, which happen to be more vulnerable to stress due to inflation [33].

We speculate that such subgroup differences in stress due to inflation may exacerbate existing health disparities. It has been reported that when inflation rises, health outcomes decline [34]. Stress may be one of the mechanisms whereby inflation leads to worse physical health outcomes.

The widespread and increasing stress due to inflation we find may have implications for mental health outcomes, practices, policies, and programs. The causal effect of stress due to inflation on mental health requires further research. Stress due to inflation may lead to poor mental health [35] through several mechanisms. A large amount of literature has shown that other macroeconomic phenomena such as recessions can cause poor mental health [36,37,38], including high rates of anxiety, depression, or suicidal ideation [36,39]. At the microlevel, a systematic review concluded that financial hardships such as low income, low assets, debt, were strongly associated with depression [40]. Policy responses should address the complexity of stress responses to multiple external threats, such as COVID and inflation.

A noteworthy finding in our study is that individuals report changing behaviors to adapt to higher prices, for example, by eating out less, eating less meat and fresh produce and working additional jobs. At the same time, coping with inflation is constrained because some expenses are to meet basic needs (e.g., food, housing). Our results are consistent with media reports that inflation may change food selection, such as cutting down on meat [41] or stretching pots of spaghetti [42]. In addition, some coping mechanisms may have negative effects on health. We found that among working-age adults in the US, one in four delayed medical treatment, which has implications for a possible cascading effect on negative health outcomes. The Inflation Reduction Act of 2022 has provided some relief by lowering health-care costs for some families and involves protections against spiraling drug costs, including penalties to Medicare if the pharmaceutical companies increase prices faster than inflation [43]. However, it is unclear whether these policies can provide relief to the families experiencing stress due to inflation.

Never before in the history of the US has the population been confronted with a pandemic and increasing inflation. Although inflation has declined, it is not going to return to the 2% level within the year [44] and price levels remain high compared to pre-pandemic levels. With increasing stress due to inflation, there is the risk that some individuals feel they are not able to manage their stress and maladaptive behaviors may occur (e.g., substance use, depression), which often leads to poor health [45]. Given how widespread stress due to inflation is, especially among economically disadvantaged individuals, stress due to inflation requires attention by researchers and policymakers alike. In particular, its likely impacts on mental and physical health require further research.

## 5. Strengths and Limitations

This study has strengths and limitations. The study is timely and used near real-time data that can be used for taking the “pulse” of the US in terms of stress due to inflation. This study was only able to determine the prevalence of stress due to inflation and its increase over time, without establishing if stress due to inflation has become chronic at the individual or household level. In addition, several factors that could potentially affect stress due to inflation were not analyzed, including cumulative lifetime stressors such as chronic poverty. Further, we did not conduct an analysis of anxiety and depression as potential drivers or consequences of stress due to inflation. Therefore, a large-scale longitudinal study with the inclusion of more potential causes and effects of stress due to inflation and other relevant stressors is warranted. Effects include coping strategies, especially potentially detrimental ones, such as delaying health care. Finally, our data have limitations. We only had data over a ten-month interval; therefore, we were unable to determine long-term trends in stress due to inflation. While we used weighting adjustments, which can help mitigate nonresponse bias in HPS, like in any research using HPS data, we cannot be sure that there is no bias in the estimates [46].

## 6. Conclusions

Stress due to inflation is remarkably common in the US among the working-age population overall and among gender and individuals that may experience other stressors such as low income, employment income loss, and COVID in particular. Despite a declining national inflation rate in recent months, stress due to inflation became significantly more prevalent between September 2022 and June 2023, suggesting that price increases may have cumulative effects on stress over time. The causes and the consequences of stress due to inflation warrant further research and policy attention. In particular, there is a need to focus on health policies in addition to anti-inflationary monetary and fiscal policies due to inflation’s potential effects on mental and physical health through stress.

## Figures and Tables

**Table 1:** Characteristics of working-age adults (18–64 years) in the United States reporting price increase in their area in the last two months in the Household Pulse Survey, September 2022–June 2023.

		14–26 September 2022		7–19 June 2023		*p*
		N	Col wt %	N	Col wt %	
ALL		32,579	100.0	38,229	100.0	
**Stress due to Inflation**						<0.0001
	Very stressed	13,666	49.4	16,713	50.6	
	Moderately stressed	9456	27.5	11,636	28.2	
	Little stressed	7327	18.2	8047	17.5	
	Not stressed	2130	4.9	1833	3.6	
**Stress due to Inflation**						<0.0001
	Very/moderately stressed	23,122	76.9	28,349	78.9	
	Little/no stress	9457	23.1	9880	21.1	
**Gender**						0.011
	Female	17,984	50.0	21,775	49.4	
	Male	13,954	47.6	15,552	47.7	
	Transgender	641	2.4	902	2.8	
**Age**						<0.0001
	18–34 Years	7640	32.6	8887	33.3	
	35–44 Years	8517	22.5	10,233	23.4	
	45–54 Years	7736	21.5	9307	21.1	
	55–64 Years	8686	23.4	9802	22.2	
**Race and Ethnicity**						0.001
	NHW	24,106	59.2	27,046	57.4	
	NHB	2265	11.3	3306	11.8	
	Hispanic	3135	19.1	3892	20.3	
	NHA	1565	5.3	2068	5.5	
	Other race	1508	5.0	1917	5.0	
**Education**						0.032
	Less than HS	691	7.0	885	7.3	
	HS	3740	29.8	4891	29.5	
	Some college	7032	22.2	8204	21.0	
	Associate degree	3462	9.3	4183	9.6	
	College	17,654	31.7	20,066	32.7	
**Lost Employment Income**						0.235
	Yes	3292	13.5	4011	13.9	
	No	29,205	86.2	34,129	85.8	
**Poverty Status (in FPL)**						0.056
	<100%	2744	12.7	3163	12.7	
	100–<200%	3974	16.4	5027	16.5	
	200–<400%	7461	22.8	9349	23.8	
	>=400%	13,688	29.8	15,078	28.0	
**Food Affordability**						0.125
	Not afford	7834	29.6	9886	31.0	
	Afford	4905	16.5	5715	16.6	
	Sufficient	18,661	48.6	21,273	47.6	
**Health Insurance**						0.098
	Yes	27,792	79.7	32,418	78.8	
	No	1682	7.7	1940	7.5	
**Marital Status**						0.330
	Married	18,277	53.6	21,537	53.9	
	Widowed	717	2.1	824	1.8	
	Separated	4514	11.2	5354	10.9	
	Divorced	606	2.4	732	2.2	
	Never married	8374	30.2	9684	30.8	
**Region**						0.002
	Northeast	4745	16.8	5299	16.7	
	South	10,371	38.3	12,881	39.4	
	Midwest	7071	20.7	8271	20.0	
	West	10,392	24.2	11,778	23.9	
**COVID**						<0.001
	Long COVID	4940	15.9	6617	17.9	
	Acute COVID	11,789	35.8	16,053	40.2	
	No COVID	15,697	47.9	15,309	41.3	

Notes: Based on 70,808 working–age adults (18–64 years) who reported price increases in the area where they live and shop, with no missing data on stress due to price increase. Missing data in marital status, lost employment income, poverty status, food affordability, health insurance, and COVID are not included in the table. The Rao–Scott chi-squared test was used to determine significant group differences for week 49 (14–26 September 2022) and week 58 (7–19 June 2023). FPL: federal poverty line; HS: high school; NHA: non-Hispanic Asian; NHB: non-Hispanic black; NHW: Non-Hispanic white; p: chi-squared probability; Wt: weighted.

**Table 2:** Characteristics of working-age adults (18–64 years) in the United States by Stress due to inflation. Household Pulse Survey, September 2022 and June 2023.

		Very Stressed	Moderately Stressed	A Little Stressed	No Stress	
ALL		50.0	27.9	17.9	4.3	
**Gender**						<0.0001
	Female	53.7	27.2	16.1	3.0	
	Male	45.5	28.8	20.0	5.7	
	Transgender	62.7	23.1	11.3	2.8	
**Age**						<0.0001
	18–34 Years	48.4	30.9	17.5	3.2	
	35–44 Years	52.7	26.3	16.7	4.2	
	45–54 Years	53.0	25.7	17.4	3.9	
	55–64 Years	46.7	26.9	20.0	6.3	
**Race and Ethnicity**						<0.0001
	NHW	46.5	29.5	19.4	4.6	
	NHB	58.8	22.5	14.4	4.3	
	Hispanic	56.8	25.8	14.3	3.1	
	NHA	37.9	32.0	23.6	6.5	
	Other	56.3	25.2	15.3	3.2	
**Education**						<0.0001
	Less than HS	64.0	19.4	13.5	3.0	
	HS	59.6	24.2	13.3	2.9	
	Some college	53.8	28.8	14.8	2.7	
	Associate degree	54.8	26.6	14.7	3.8	
	College	34.1	32.8	26.0	7.1	
**Lost Employment Income**						<0.0001
	Yes	70.8	18.6	9.1	1.5	
	No	46.6	29.3	19.3	4.7	
**Poverty Status (in FPL)**						<0.0001
	<100%	68.8	19.6	10.0	1.6	
	100–<200%	64.7	24.5	9.6	1.2	
	200–<400%	51.4	29.5	16.1	3.0	
	>=400%	29.9	32.3	28.8	8.9	
**Food Affordability**						<0.0001
	Cannot afford	78.6	17.8	3.5	0.2	
	Afford	59.3	28.9	11.1	0.7	
**Health Insurance**						<0.0001
	Yes	48.2	28.7	18.5	4.7	
	No	61.4	21.8	14.2	2.6	
**Marital Status**						<0.0001
	Married	47.5	28.1	19.4	5.0	
	Widowed	61.8	23.0	12.0	3.2	
	Separated	59.0	23.5	14.1	3.4	
	Divorced	65.4	19.8	13.1	1.6	
	Never married	49.0	29.9	17.3	3.7	
**Region**						<0.0001
	Northeast	47.1	28.6	19.3	5.0	
	South	53.0	26.4	16.5	4.1	
	Midwest	47.9	29.0	18.6	4.5	
	West	48.9	28.7	18.3	4.0	
**COVID**						<0.0001
	Long COVID	61.6	24.7	12.1	1.6	
	Acute COVID	43.7	29.9	21.4	5.0	
	No COVID	50.8	27.4	17.1	4.7	
**COVID-19 Vaccination**						<0.0001
	Yes	46.5	28.9	19.9	4.8	
	No	63.5	24.0	10.1	2.4	

Note: Based on 70,808 working-age adults (18–64 years) who reported price increases in the area where they live and shop, with no missing data on stress due to price increase. Missing data in paying for household expenses, marital status, lost employment income, poverty status, food affordability, health insurance, COVID, and COVID-19 vaccination are not included in the table. The Rao–Scott chi-squared test was used to determine significant group differences by stress of price changes in the last two months. COVID: coronavirus disease; FPL: federal poverty line; HS: high school; NHA: non-Hispanic Asian; NHB: non-Hispanic black; NHW: non-Hispanic white; p: chi-squared probability Wt: weighted.

**Table 3:** Cumulative odds ratios (CORs) and 95% confidence intervals (CIs) from ordinal logistic regression on stress due to inflation in working-age adults (18–64 years), Household Pulse Survey, September 2022 and June 2023.

		COR	95% CI	*p*
**Week**				
	14–26 September 2022 (Ref)			
	7–19 June 2023	1.08	[1.02, 1.14]	0.013
**Gender**				
	Female	1.34	[1.27, 1.42]	<0.0001
	Male (Ref)			
	Transgender	1.46	[1.22, 1.75]	<0.0001
**Age**				
	18–34 Years (Ref)			
	35–44 Years	1.10	[1.01, 1.19]	0.021
	45–54 Years	1.03	[0.95, 1.12]	0.417
	55–64 Years	0.77	[0.71, 0.84]	<0.0001
**Race and Ethnicity**				
	NHW (Ref)			
	NHB	1.11	[0.99, 1.25]	0.086
	Hispanic	1.09	[0.99, 1.21]	0.079
	NHA	1.00	[0.88, 1.13]	0.996
	Other	1.08	[0.95, 1.23]	0.240
**Education**				
	Less than HS	1.24	[1.01, 1.52]	0.040
	HS	1.47	[1.35, 1.61]	<0.0001
	Some college	1.32	[1.24, 1.40]	<0.0001
	Associate degree	1.42	[1.29, 1.55]	<0.0001
	College (Ref)			
**Lost Employment Income**				
	No (Ref)			
	Yes	1.72	[1.53, 1.94]	<0.0001
**Poverty Status (in FPL)**				
	<100%	1.87	[1.62, 2.16]	<0.0001
	100–<200%	1.89	[1.72, 2.09]	<0.0001
	200–<400%	1.52	[1.41, 1.65]	<0.0001
	>=400% (Ref)			
**Food Affordability**				
	Not afford	7.14	[6.58, 7.74]	<0.0001
	Afford (Ref)			
	Sufficient	3.08	[2.84, 3.34]	<0.0001
**Health Insurance**				
	Yes (Ref)			
	No	0.90	[0.77, 1.05]	0.161
**Marital Status**				
	Married (Ref)			
	Widowed	1.04	[0.85, 1.28]	0.678
	Separated	0.99	[0.89, 1.09]	0.814
	Divorced	0.97	[0.75, 1.26]	0.836
	Never married	0.77	[0.71, 0.84]	<0.0001
**Region**				
	Northeast (Ref)			
	South	1.03	[0.94, 1.13]	0.551
	Midwest	0.95	[0.87, 1.05]	0.311
	West	1.01	[0.92, 1.11]	0.854
**COVID**				
	Long COVID	1.46	[1.32, 1.62]	<0.0001
	Acute COVID	1.10	[1.03, 1.17]	0.003
	No COVID (Ref)			
**COVID-19 Vaccination**				
	Yes	0.71	[0.65, 0.77]	<0.0001
	No (Ref)			

Notes: Based on 70,808 working-age adults (18–64 years) who reported price increases in the area where they live and shop, with no missing data on stress due to price increase. Estimates for missing data in marital status, lost employment income, poverty status, food affordability, health insurance, COVID, and COVID-19 vaccination are not included in the table. Intercepts for high stress, moderate stress, and little stress are not presented. COVID: coronavirus disease; FPL: Federal poverty line; Ref: reference group.

**Table 4:** Plans and strategies to cope with price increase working-age adults (18–64 years) in the United States Census Household Pulse Survey, September 2022–June 2023.

		14–26 September 2022	7–19 June 72023	*p*
		
**Shop**				<0.0001
	Shop with low-price coupons	63.8	68.5	
	No	36.2	31.5	
**Eat out**				0.045
	Eating out less	58.1	59.4	
	No	41.9	40.6	
**Purchases**				<0.0001
	Delay major purchases	51.8	54.4	
	No	48.2	45.6	
**Brand to generics**				0.001
	Switch from brands to generics	48.2	50.6	
	No	51.8	49.4	
**Subscriptions**				<0.0001
	Cancel/reduce subscriptions	40.3	45.3	
	No	59.7	54.7	
**Events**				0.295
	Cancel/decrease events	39.9	40.6	
	No	60.1	59.4	
**Savings**				0.418
	Less savings	36.7	36.1	
	No	63.3	63.9	
**Meat and fresh produce**				0.016
	Less meat and fresh produce	37.0	35.6	
	No	63.0	64.4	
**Transportation**				<0.0001
	Drive less/change mode	34.3	24.6	
	No	65.7	75.4	
**Utilities**				0.309
	Decrease use utilities	25.5	24.8	
	No	74.5	75.2	
**Credit card use**				<0.0001
	Increase use of credit	28.8	24.8	
	No	71.2	75.2	
**Medical treatment**				0.005
	Delay medical treatment	24.2	26.1	
	No	75.8	73.9	
**Additional jobs**				<0.0001
	Work additional jobs	20.5	25.7	
	No	79.5	74.3	
**Help**				0.063
	Ask friends/family for help	15.6	14.6	
	No	84.4	85.4	
**No change**				0.005
	Made no changes	7.3	6.3	
	No	92.7	93.7	
**Charity**				0.001
	Utilize benefits from charities	5.2	6.1	
	No	94.8	93.9	
**Other**				0.048
	Yes	4.4	5.1	
	No	95.6	94.9	
**Housing**				<0.0001
	Move to less expensive housing	3.9	5.9	
	No	96.1	94.1	
**Childcare**				<0.0001
	Change/reduce childcare plans	4.2	5.8	
	No	95.8	94.2	

Notes: Based on 70,808 working-age adults (18–64 years) who reported price increases in the area where they live and shop, with no missing data on stress due to price increase. Wt: weighted.

## Data Availability

No restrictions apply to the availability of these data. Data were obtained from the US Census Bureau and are available at https://www.census.gov/programs-surveys/household-pulse-survey/datasets.html (accessed on 11 January 2024).

## References

[1] Current US Inflation Rates: 2000–2023. https://www.usinflationcalculator.com/inflation/current-inflation-rates/.

[2] U.S. Inflation Rate 1960–2023. https://www.macrotrends.net/countries/USA/united-states/inflation-rate-cpi.

[3] Monthly Inflation Rate U.S. 2023. Statista. https://www.statista.com/statistics/273418/unadjusted-monthly-inflation-rate-in-the-us/.

[4] Wu C., Louie P., Bierman A., Schieman S. (2023). Assessment of Sociodemographics and Inflation-Related Stress in the US. JAMA Netw. Open.

[5] Stress in America 2022: Concerned for the Future, Beset by Inflation. https://www.apa.org/news/press/releases/stress/2022/concerned-future-inflation.

[6] (2023). Mental Health and Inflation: Understanding the Impact. GoodTherapy.org Therapy Blog. https://www.goodtherapy.org/blog/inflation-and-mental-health/.

[7] Epstein Y.M., Babad E.Y. (1982). Economic Stress: Notes on the Psychology of Inflation. J. Appl. Soc. Psychol..

[8] Agorastos A., Chrousos G.P. (2022). The neuroendocrinology of stress: The stress-related continuum of chronic disease development. Mol. Psychiatry.

[9] Wheaton B., Young M., Montazer S., Stuart-Lahman K., Aneshensel C.S., Phelan J.C., Bierman A. (2013). Social Stress in the Twenty-First Century. Handbook of the Sociology of Mental Health. Handbooks of Sociology and Social Research.

[10] Converse M., Curtin R.T., Kallick M. (1980). Coping with inflation. Economic Outlook USA.

[11] Epel E., Crosswell A.D., Mayer S.E., Prather E.A., Slavich G.M., Puterman E., Berry Mendes W. (2018). More than a feeling: A unified view of stress measurement for population. Front. Neuroendocrinol..

[12] Bhattarai A., Stein J. Inflation Is falling. Why Aren’t People Noticing? Washington Post, 13 April 2023. https://www.washingtonpost.com/business/2023/04/12/inflation-cpi-still-high/.

[13] Pfajfar D., Santoro E. (2013). News on Inflation and the Epidemiology of Inflation Expectations. J. Money Credit. Bank..

[14] Doepke J., Dovern J., Fritsche U., Slacalek J. (2008). The Dynamics of European Inflation Expectations. BE J. Macroecon..

[15] Curtin R. (2010). Inflation expectations and empirical tests. Inflation Expectations.

[16] Ehrmann M., Pfajfar D., Santoro E. Consumers’ Attitudes and Their Inflation Expectations. FEDS Working Paper No. 2015-015. https://ssrn.com/abstract=2595719.

[17] Bureau U.C. Week 50 Household Pulse Survey: 5 October–17 October. Census.gov. https://www.census.gov/data/tables/2022/demo/hhp/hhp50.html.

[18] Smith S.M., Edwards R., Duong H.C. Unemployment Rises in 2020, as the Country Battles the COVID-19 Pandemic: Monthly Labor Review: U.S. Bureau of Labor Statistics. https://www.bls.gov/opub/mlr/2021/article/unemployment-rises-in-2020-as-the-country-battles-the-covid-19-pandemic.htm.

[19] How to Stop Living Paycheck to Paycheck. US News & World Report. https://money.usnews.com/credit-cards/articles/how-many-americans-are-living-paycheck-to-paycheck.

[20] How Big Is the Generational Wealth Gap in America?|Self. https://www.self.inc/info/generational-wealth-gap/.

[21] US Census Bureau (2022). Measuring Household Experiences during the Coronavirus Pandemic. Census.gov. https://www.census.gov/householdpulsedata.

[22] Thurner C., Stengel A. (2023). Long-COVID syndrome: Physical–mental interplay in the spotlight. Inflammopharmacology.

[23] National Center for Health Statistics U.S. Census Bureau, Household Pulse Survey, Phase 3.8. Long COVID. https://www.cdc.gov/nchs/covid19/pulse/long-covid.htm.

[24] Khan W., Khan B.M., Yasen S., Al-Dahiri A., Al-Jumeily D., Dajani K., Hussain A. (2022). COVID-19 Vaccination and Mental Stress within Diverse Sociodemographic Groups. Int. J. Environ. Res. Public Health.

[25] Rust K. (1985). Variance Estimation for Complex Estimators in Sample Surveys—ProQuest. J. Off. Stat..

[26] (2023). Food Inflation Is Easing—But Price Breaks Are Few and Far between. https://www.cbsnews.com/news/inflation-groceries-where-prices-are-falling-2023/.

[27] LendingClub Corporation The Number of Consumers Living Paycheck to Paycheck Has Increased Year-Over-Year across All Income Levels. https://www.prnewswire.com/news-releases/the-number-of-consumers-living-paycheck-to-paycheck-has-increased-year-over-year-across-all-income-levels-301596552.html.

[28] Bruine de Bruin W., van der Klaauw W., Downs J.S., Fischhoff B., Topa G., Armantier O. (2010). The Effect of Question Wording on Reported Expectations and Perceptions of Inflation. Rochester, NY, USA. https://papers.ssrn.com/abstract=1594890.

[29] Bryan M.F., Venkatu G. The Curiously Different Inflation Perspectives of Men and Women. Economic Commentary, 1 November 2001. https://www.clevelandfed.org/publications/economic-commentary/2001/ec-20011101-the-curiously-different-inflation-perspectives-of-men-and-women.

[30] Müller H., Schmidt T., Rieger J., Hornig N., Hufnagel L.M. The Inflation Attention Cycle: Updating the Inflation Perception Indicator (IPI) up to February 2023. A Research Note. TU Dortmund University, Dortmund Center for Data-Based Media Analysis (DoCMA). 2023. Report No.: 13. https://econpapers.repec.org/paper/zbwdocmaw/13.htm.

[31] Ma N., Siu Y.W., Cheong T.S., Tung B. (2022). Impact of COVID-19 on lifestyle and financial behaviour: The implications to research in financial vulnerability. Front. Psychol..

[32] Barrett P., Das S., Magistretti G., Pugacheva E., Wingender P. (2023). Long COVID? Prospects for economic scarring from the pandemic. Contemp. Econ. Policy.

[33] Andrade C., Gillen M., Molina J.A., Wilmarth M.J. (2022). The Social and Economic Impact of COVID-19 on Family Functioning and Well-Being: Where do we go from here?. J. Fam. Econ. Issues.

[34] Duffy S. When Inflation Rises, Health Outcomes Fall. Harvard Business Review, 29 November 2022. https://hbr.org/2022/11/when-inflation-rises-health-outcomes-fall.

[35] High Inflation Rates Impact Almost Every Aspect of Our Lives, Including Mental Health. Verywell Mind. https://www.verywellmind.com/how-rising-inflation-is-impacting-mental-health-5546955.

[36] Wilkinson L.R. (2016). Financial Strain and Mental Health among Older Adults during the Great Recession. J. Gerontol. Ser. B Psychol. Sci. Soc. Sci..

[37] Glei D.A., Goldman N., Weinstein M. (2019). A growing socioeconomic divide: Effects of the Great Recession on perceived economic distress in the United States. PLoS ONE.

[38] Córdoba-Doña J.A., Escolar-Pujolar A., San Sebastián M., Gustafsson P.E. (2016). How are the employed and unemployed affected by the economic crisis in Spain? Educational inequalities, life conditions and mental health in a context of high unemployment. BMC Public Health.

[39] Haw C., Hawton K., Gunnell D., Platt S. (2015). Economic recession and suicidal behaviour: Possible mechanisms and ameliorating factors. Int. J. Soc. Psychiatry.

[40] Guan N., Guariglia A., Moore P., Xu F., Al-Janabi H. (2022). Financial stress and depression in adults: A systematic review. PLoS ONE.

[41] Han Z. Inflation Is Forcing Americans to Change Their Diets: “We Make Vegetable Soup”. 2022. MarketWatch. https://www.marketwatch.com/story/inflation-is-forcing-americans-to-change-their-diets-11655932733.

[42] Hughes T. (2022). “It Didn’t Feel Like $80 Worth of Food”: Inflation Is Making It Hard to Make Healthy Food Choices. USA Today. https://www.usatoday.com/story/news/nation/2022/04/24/inflation-means-higher-food-prices/7284908001/?gnt-cfr=1.

[43] Levitt L. (2022). The Inflation Reduction Act Is a Foot in the Door for Containing Health Care Costs. JAMA Health Forum.

[44] Department AHIWH The US Economy’s Inflation Challenge. IMF..

[45] Harris A., Smith T. (2022). Monetary Sanctions as Chronic and Acute Health Stressors: The Emotional Strain of People Who Owe Court Fines and Fees. RSF Russell Sage Found. J. Soc. Sci..

[46] Peterson S., Toribio N., Farber J. (2021). Nonresponse Bias Report for the 2020 Household Pulse Survey. 24 March 2021. U.S. Census Bureau. https://www2.census.gov/programs-surveys/demo/technical-documentation/hhp/2020_HPS_NR_Bias_Report-final.pdf.

